# Forecasting dengue and influenza incidences using a sparse representation of Google trends, electronic health records, and time series data

**DOI:** 10.1371/journal.pcbi.1007518

**Published:** 2019-11-21

**Authors:** Prashant Rangarajan, Sandeep K. Mody, Madhav Marathe

**Affiliations:** 1 Departments of Computer Science and Mathematics, Birla Institute of Technology and Science, Pilani, India; 2 Department of Mathematics, Indian Institute of Science, Bangalore, India; 3 Department of Computer Science, Network, Simulation Science and Advanced Computing Division, Biocomplexity Institute, University of Virginia, Charlottesville, Virginia, United States of America; Los Alamos National Laboratory, UNITED STATES

## Abstract

Dengue and influenza-like illness (ILI) are two of the leading causes of viral infection in the world and it is estimated that more than half the world’s population is at risk for developing these infections. It is therefore important to develop accurate methods for forecasting dengue and ILI incidences. Since data from multiple sources (such as dengue and ILI case counts, electronic health records and frequency of multiple internet search terms from Google Trends) can improve forecasts, standard time series analysis methods are inadequate to estimate all the parameter values from the limited amount of data available if we use multiple sources. In this paper, we use a computationally efficient implementation of the known variable selection method that we call the Autoregressive Likelihood Ratio (ARLR) method. This method combines sparse representation of time series data, electronic health records data (for ILI) and Google Trends data to forecast dengue and ILI incidences. This sparse representation method uses an algorithm that maximizes an appropriate likelihood ratio at every step. Using numerical experiments, we demonstrate that our method recovers the underlying sparse model much more accurately than the lasso method. We apply our method to dengue case count data from five countries/states: Brazil, Mexico, Singapore, Taiwan, and Thailand and to ILI case count data from the United States. Numerical experiments show that our method outperforms existing time series forecasting methods in forecasting the dengue and ILI case counts. In particular, our method gives a 18 percent forecast error reduction over a leading method that also uses data from multiple sources. It also performs better than other methods in predicting the peak value of the case count and the peak time.

## Author summary

Dengue and influenza-like illness (ILI) are leading causes of viral infection in the world and hence it is important to develop accurate methods for forecasting their incidence. We use Autoregressive Likelihood Ratio method, which is a computationally efficient implementation of the variable selection method, in order to obtain a sparse (non-lasso) representation of time series, Google Trends and electronic health records (for ILI) data. This method is used to forecast dengue incidence in five countries/states and ILI incidence in USA. We show that this method outperforms existing time series methods in forecasting these diseases. The method is general and can also be used to forecast other diseases.

## Introduction

Dengue is a mosquito-borne viral disease that affects a large fraction of the world [1]. It is estimated [2] that almost half the world’s population spread out over 128 countries is at risk of dengue infection while 400 million people could actually be infected by dengue [3] every year. A large fraction of these cases occur in low income countries. Of these, about 100 million are estimated [3] to exhibit clinical symptoms. In the past decade, dengue cases have also been reported in Europe, China, and the USA [1] thus expanding the regions that could witness dengue outbreaks even further.

Influenza is another viral disease that affects a significant fraction of the world population. It is estimated that 3 to 5 million people worldwide are afflicted with severe illness due to influenza-like illness (ILI) of whom between 300,000 to 650,000 die [4]. Deaths occur mainly among people aged 65 years or above in the developed world [5] and children below 5 years of age in developing countries [6].

Given the huge social, economic, and health burden of dengue and ILI, it is important to be able to accurately forecast dengue and ILI incidences. Such forecasts would permit timely and adequate deployment of experienced medical personnel such as physicians and nurses, resources such as mosquito nets and antivirals (especially, flu vaccines in the case of Influenza A and B), and timely application of emergency vector control measures in the affected regions/countries. Such measures can reduce mortality rates in the case of severe dengue from more than 20% to less than 1% [1]. In the case of influenza, a recent study [7] estimated that vaccinated adults were up to 80% less likely to die than unvaccinated flu-hospitalized patients.

Several methods have been proposed to forecast dengue incidence. Some of these methods were developed in the context of the Dengue Forecasting Project [8]. One class of methods uses deterministic differential equations and primarily focuses on dengue transmission [9]. Such methods are reviewed in [10]. Another class of models follows a data-driven approach and uses techniques such as machine learning [11, 12]. Other examples in this class include seasonal autoregressive models that incorporate weather information [13–19] and hybrid models [20].

Another line of approach uses Internet searches, social media activity and phone data to forecast dengue and ILI incidences [21–30]. Other recent approaches for forecasting disease outbreaks use a variety of methods such as data-driven agent-based models [31], ensemble methods [32], phenomenological models [33], support vector machines [34], superensemble methods [35–38], neural networks [39], spatio-temporal methods [40] and delta densities [41]. A comparison of several of these methods can be found in [42]. A recent leading method for forecasting dengue and ILI incidences is AutoRegression with General Online data (ARGO) [43, 44] that combines autoregressive processes with Google Trends and other online data.

### Summary of contributions and significance

In this paper, we focus on a time series modelling approach to forecast dengue and ILI incidences. The standard time series methods for fitting models such as autoregressive models yield dense models. In other words, most of the regressor coefficients (model parameters) are non-zero. However, in the context of forecasting dengue and ILI incidences, it is important to have sparse models representing the data. There are several reasons for this. Using only the past incidence data to forecast future values may not be adequate since the forecast accuracies may not be high [43, 44]. It should be added that this need not always be the case [45, 46]. In general, one hopes [38, 46, 47] to improve forecast accuracies by including additional sources of data such as frequencies of search terms from Google Trends data (www.google.com/trends) and electronic health records. Each such additional source of data leads to additional parameters that need to be estimated from the data. However, the amount of data available is typically insufficient to robustly estimate the required parameter values. Hence we need to represent this data using sparse models. A standard method for obtaining sparse models is the lasso method [48, 49]. The lasso method has been implemented in the ARGO method [43, 44] for forecasting dengue and ILI incidences. We implement a computationally efficient method for variable selection, in order to obtain sparse representations of the data, that outperforms the lasso method. This is demonstrated by fitting autoregressive models using both the lasso method and our method to synthetic time series data generated from sparse models. We find that our method recovers the underlying sparse model with much greater accuracy than the lasso method.

We apply the method for fitting a sparse vector autoregressive model to dengue and ILI case counts time series data. Further, we adopt a comprehensive method to remove the seasonal component before applying the regression model. For both dengue and ILI, we use exactly the same data as was used for investigating the ARGO method [43, 44]. This data has been made publicly available by authors of the ARGO method and this facilitates direct comparison of our method with the ARGO and other methods.

For dengue, we analyze monthly aggregated dengue case count data from five countries/states: 3 in Asia (Singapore, Taiwan, and Thailand) and 2 in South America (Brazil and Mexico). This is combined with the top ten queries that were most highly correlated with the term ‘dengue’ in each country [43] using Google Trends data. For each country, the monthly aggregated search fractions of these terms [43] were then used. In the case of ILI, we used weekly ILI case count data from the United States. This is combined with electronic health records and Google Trends data [44].

The combination of the sparse representation technique and multiple data sources that we use yields a forecasting method that outperforms other competing methods in terms of forecast error measures. More specifically, our method achieves an average of 18% reduction in the forecast error over the ARGO method which is a leading method that also uses data from multiple sources. Our method is general and could also be used to forecast other disease outbreaks.

## Materials and methods

We describe different methods that can be used to forecast dengue and ILI incidences. For our method, we first describe the general method applicable to any time series data. Subsequently, we detail the additional preprocessing steps required to process the dengue and ILI data. The other methods that are described include ARGO, Glmnet lasso, Kalman filtering, ensemble, and the naive method.

### Autoregressive Likelihood Ratio (ARLR) method

As mentioned in the introduction, it is important to develop sparse models to represent the data given the large number of data sources (such as dengue or ILI case counts and frequency of multiple Internet search queries from Google Trends) and the limited amount of data available from each source (especially dengue and ILI case counts) for training the model. In this section, we describe a computationally efficient method for obtaining sparse vector autoregressive models from multivariate time series data.

Let the observed time series data *y* be represented by an *N* × *k* matrix:

$$\begin{array}{c} y=\{y_{t}^{\left(j\right)},j=1,2,\ldots k;\;t=1,2,\ldots N\} \end{array}$$ (1)

Here *N* stands for the number of data points and *k* is the number of variables.

Following standard time series modelling methods [50] we model this observed data *y* by a zero-mean, weakly stationary Vector Autoregressive (VAR) process satisfying the following equation:

$$\begin{array}{c} V_{t}=A_{1}V_{t-1}+A_{2}V_{t-2}+\ldots +A_{p}V_{t-p}+\epsilon_{t} \end{array}$$ (2)

Here $V_{t}={\left\{V_{t}^{\left(1\right)},\ldots ,V_{t}^{\left(k\right)}\right\}}^{T}$ is a column vector of variables that model the data. This equation expresses *Y* at time *t* in terms of its own values at previous times (lagged values) up to time *t* − *p*. Here *p* is called the order of the model and is also the maximum lag. Each of *A*_1_, *A*_2_, … *A*_*p*_ is a *k* × *k* real coefficient matrix and *ϵ*_*t*_ is *k* × 1 normally distributed white noise with zero mean and constant covariance matrix:

$$\begin{array}{c} E\left(\epsilon_{t}\epsilon_{t}^{T}\right)=\Sigma_{\epsilon } \end{array}$$ (3)

Standard methods for estimating coefficients for the above VAR model use either linear regression or the Yule-Walker equations [50]. The models obtained using such methods are dense in the sense that the coefficient matrices *A*_*i*_ are all dense wherein most (if not all) of the matrix entries are non-zero. In the context of forecasting dengue or ILI incidence, using such time series models becomes problematic since this large number of parameters need to be estimated from a limited amount of data leading to noisy or inaccurate estimates. This problem is further compounded if we also incorporate Google Trends or electronic health records data into the modeling process, further increasing the number of parameters to be estimated. A standard way of overcoming this problem is to use sparse models [43, 44, 48, 49].

In this paper, we use an alternative sparse modelling method, a variable selection method, that is already known [51]. Variable selection method, however, is computationally inefficient. We have devised a computationally efficient process for variable selection. Variable selection is based on comparing the relative likelihoods of candidate solutions [51]. Instead of including, at one go, all variables at all possible lags as predictors for each of the response variables, each variable at each individual lag is included (removed) as a predictor, one at a time, based on the size of the observed error in the response variable(s) before and after the inclusion (removal). A detailed description of the method is given in the Supporting Information (S1 Text). To briefly summarize, starting from a suitable initial VAR model (typically the trivial model), lagged predictor variables are added or removed depending on whether their inclusion (exclusion) significantly increases (decreases) the likelihood that the data is explained by the model, respectively. This process is continued until no further variables can be added or removed.

As mentioned above, we have implemented the variable selection method in a computationally efficient manner described in the Supporting Information (S1 Text). We call this the Autoregressive Likelihood Ratio (ARLR) method. The efficiency of our algorithm is demonstrated using time complexity calculations in the Supporting Information (S1 Text). We also use an alternative stopping criterion given by the *AICC* (corrected AIC) criterion [52]. Both these factors enable this method to effectively handle thousands of variables. The computationally efficient method is general and is applicable beyond disease forecasting.

We now describe the preprocessing steps required in order to make the dengue/influenza data amenable to analysis by our method. We model the time series data for each country/state as given below following a systematic approach. Given the original data *C*_*t*_ (dengue or ILI case count), we first separate out the deterministic and stochastic components (*F*_*t*_ and *U*_*t*_, respectively). In order to accomplish this, we determine whether the model to be used is additive (*C*_*t*_ = *H*_*t*_ + *U*_*t*_) or multiplicative (*C*_*t*_ = *H*_*t*_*U*_*t*_) by computing the mean and standard deviation in a fixed-length window that slides over the data. If the underlying model is additive, the mean remains approximately constant across the windows whereas if the underlying model is multiplicative, it is the ratio of the mean to the standard deviation that remains approximately constant. We find the latter to be the case and hence we choose a multiplicative model. In the case of Taiwan, we find that it is log(*C*_*t*_), rather than *C*_*t*_, which follows the multiplicative model. Consequently, in the case of Taiwan, we take log(*C*_*t*_) to be the original data so that the subsequent analysis is identical for all cases.

The multiplicative model can be transformed to a new additive model by taking a logarithm: log(*C*_*t*_) = log(*H*_*t*_) + log(*U*_*t*_). Letting *Y*_*t*_ = log(*U*_*t*_), *F*_*t*_ = log(*H*_*t*_) and $C_{t}^{\prime }=\text{log}\left(C_{t}\right)$, we finally get

$$\begin{array}{c} C_{t}^{\prime }=F_{t}+Y_{t}. \end{array}$$ (4)

Thus we need to additively decompose the transformed data into the deterministic component *F*_*t*_ (which is essentially comprised of a trend and a seasonal component) and the stationary component *Y*_*t*_. To do this, we first determine the seasonality (*s*) of the process as follows. We compute a smoothed power spectrum of the original forecast variable. The spectrum is smoothed by appending zeros to the case count up to a length of 1024. We obtain the location of the dominant peak. The corresponding period *s* (in either months or weeks) is taken to be the seasonality (s). Now the deterministic component is found by fitting the following simple model to the data:

$$\begin{array}{c} C_{t}^{\prime }=\mu +c_{1}C_{t-1}^{\prime }+c_{s}C_{t-s}^{\prime }+c_{2s}C_{t-2s}^{\prime }+Y_{t}. \end{array}$$ (5)

This model also accounts for a mean and second-order seasonality, if present in the data. We refer to this step as the *prefitting* step. For this prefitting step, we use the same algorithm as described in the Supporting Information (S1 Text). The residuals from this prefitting step comprise the stationary component *Y*_*t*_. This stationary component is what is used the subsequent analysis.

Let *Y*_*t*_ be obtained as above after prefitting. Here, *t* represents time in months for dengue and time in weeks for ILI. Let *X*_*t*,*g*_ be the log-transformed Google search frequency for the search term *g* at time *t*. These can be considered as exogenous terms [43]. There can be additional exogenous terms like the electronic health records in the case of ILI. These exogenous terms are represented as *Z*_*t*,*j*_ (after an appropriate transformation) for the *j*th additional exogenous term at time *t*. Then our model is formulated as given below, following [43, 44]:

$$\begin{array}{c} Y_{t}=\mu_{Y}+\sum_{m\in M}a_{m}Y_{t-m}+\sum_{g\in G}k_{g}X_{t,g}+\sum_{j\in J}q_{j}Z_{t,j}+\epsilon_{t} \end{array}$$ (6)

where *μ*_*Y*_ is the mean of the process, *M* is the set of AR lags, *G* is the set of Google query terms, *J* is the set of additional exogenous terms, and *ϵ*_*t*_ is the noise in the process which follows a zero mean Gaussian distribution. The set *G* is chosen to be the set of 10 Google search terms that are most correlated with dengue for each country/state [43] for case of dengue whereas *G* is the set of 129 Google search terms that are most correlated with ILI for the United States [44] in the case of ILI. The unknown parameters *a*_*m*_, *k*_*g*_, and (if applicable) *q*_*j*_ are estimated using the same algorithm as described in the Supporting Information (S1 Text). This method can be trivially extended to incorporate exogenous variables *X*_*t*,*g*_ (and *Z*_*t*,*j*_). Once the unknown parameters are estimated, the forecast variable *Y*_*t*+1_ for the month/week *t* + 1 can be predicted using the above equation. Subsequently, the final forecast of the dengue or ILI case count at time *t* + 1 is obtained by using Eq (5) followed by an exponential transform. The code implementing the ARLR method can be made available by the authors upon request.

#### Uncertainty quantification

We quantify the uncertainties in the ARLR forecast by using the bootstrap method described in [53]. This is the standard method that is widely used for uncertainty quantification in autoregressive modeling. Suppose we wish to obtain the uncertainty quantification of the nowcast *Y*_*T*+1_ using the observations of *Y*_*t*_ until time *T*. Using the estimated values of the parameters *a*_*m*_, *k*_*g*_, and (if applicable) *q*_*j*_ obtained through our algorithm and the observed values of *Y*_*t*_, *X*_*t*_ and *Z*_*t*_, we estimate the residuals ${\hat{\epsilon }}_{t}$ for *t* = 1, 2, …, *T* as follows:

$$\begin{array}{c} {\hat{\epsilon }}_{t}=Y_{t}-\mu_{Y}-\sum_{m\in M}a_{m}Y_{t-m}-\sum_{g\in G}k_{g}X_{t,g}-\sum_{j\in J}q_{j}Z_{t,j}. \end{array}$$ (7)

Following the standard bootstrap approach [53], we now create *T* bootstrapped residuals $\epsilon_{t}^{*}$ (*t* = 1, 2, …, *T*) by sampling the estimated residuals ${\hat{\epsilon }}_{1}$, ${\hat{\epsilon }}_{2}$, …, ${\hat{\epsilon }}_{T}$ with replacement *T* times. We now obtain the bootstrap sample $Y_{t}^{*}$ (*t* = 1, 2, …, *T*) as follows:

$$\begin{array}{c} Y_{t}^{*}=\mu_{Y}+\sum_{m\in M}a_{m}Y_{t-m}^{*}+\sum_{g\in G}k_{g}X_{t,g}+\sum_{j\in J}q_{j}Z_{t,j}+\epsilon_{t}^{*}. \end{array}$$ (8)

We now forecast $Y_{T+1}^{*}$ from the above equation. This gives a bootstrap nowcast $Y_{T+1}^{*}$. The above procedure is repeated 1000 times to obtain 1000 bootstrap nowcast values of $Y_{T+1}^{*}$. Using these 1000 bootstrap values we can now estimate the probabilities of the nowcast falling in different value bins (thereby quantifying the uncertainty) as described in the results section below.

#### Multi-step ahead forecasts

For multi-step ahead forecasts, the time indices are shifted appropriately so that observed data (both *Y* and *X*) only up to time *t* is used for making the forecasts. In addition, we also need to propagate *X* and *Z*. This is done by first stationarizing each variable in {*X*, *Z*} using the same ARLR prefitting procedure (cf. Eq (5)) with the same lags as is done for *Y*. Denote the residuals of this (diagonal) stationarizing operator by *W*. We fit an autoregressive model for the residuals *W* with additional dependence on *Y* at a small number of short lags:

$$\begin{array}{c} W_{t}=\mu_{Y}+\sum_{q\in Q}B_{q}W_{t-q}+\sum_{m\in M^{\prime }}b_{m}Y_{t-m}+\epsilon_{t} \end{array}$$ (9)

where *W*_*t*_ are the residuals obtained by stationarizing {*X*_*t*_, *Z*_*t*_} taken together, *B*_*q*_ is a the matrix coefficient of *W* at lag *q* and *b*_*m*_ is the coefficient vector multiplying the scalar forecast variable *Y*_*t*_ at lag *m*. The setup sections (see below) for dengue and ILI specify the set of lags in each of the two sets *Q* and *M*′ for the datasets under consideration.

### ARGO

ARGO [43] is a multivariate linear regression model, which consists of an autoregressive (AR) process coupled with certain exogenous variables derived from Google search queries. The Google search queries used here are those related to either dengue or ILI depending on the disease that is being forecast. The method utilises the lasso technique (described earlier) in order to obtain a sparse representation [43]. ARGO makes the reasonable assumption that there is a positive correlation between the seasons in which dengue is prevalent and dengue related Google search queries.

As before, let *Y*_*t*_ represent the dengue or ILI case counts after a logarithmic or logit transformation, let *X*_*t*,*g*_ be the log-transformed Google search frequency for the search term *g*, and *Z*_*t*,*j*_ is the *j*th additional exogenous term (after an appropriate transformation) at time *t*. Then, the ARGO model equation [43] is exactly the same as Eq (6). The unknown parameters are estimated using a variant of the lasso method by minimizing the following sum of squared errors with an added *l*_1_ regularization term:

$$\begin{array}{c} \sum_{t}(y_{t}-\mu_{y}-\sum_{m\in M}a_{m}y_{t-m}-\sum_{g\in G}k_{g}x_{t,g}-\sum_{j\in J}q_{j}z_{t,j}{)}^{2}\\ +\sum_{m\in M}\lambda_{a_{m}}|a_{m}|+\sum_{g\in G}\lambda_{k_{g}}|k_{g}|+\sum_{j\in J}\lambda_{q_{j}}\left|q_{j}\right| \end{array}$$ (10)

where *y*_*t*_, *x*_*t*,*g*_, and *z*_*t*,*j*_ represent the observed values of *Y*_*t*_, *X*_*t*,*g*_, and *Z*_*t*,*g*_, respectively. Further, $\lambda_{a_{m}}$, $\lambda_{k_{g}}$, and $\lambda_{q_{j}}$ are the lasso regularization parameters. The unknowns in this model, *μ*_*Y*_, *a*_*m*_, *k*_*g*_, *q*_*j*_ can be estimated from this equation by minimizing it with respect to these parameters [43]. Once the model parameters are known, the model can be used to predict the disease case counts for the next time point.

### Glmnet lasso

The Glmnet lasso method [48] is identical to ARGO method except that the unknown parameters are estimated using the standard lasso method by minimizing the following sum of squared errors with an added *l*_1_ regularization term:

$$\begin{array}{c} \sum_{t}(y_{t}-\mu_{y}-\sum_{m\in M}a_{m}y_{t-m}-\sum_{g\in G}k_{g}x_{t,g}-\sum_{j\in J}q_{j}z_{t,j}{)}^{2}\\ +\lambda_{a}\sum_{m\in M}|a_{m}|+\lambda_{k}\sum_{g\in G}|k_{g}|+\lambda_{q}\sum_{j\in J}\left|q_{j}\right| \end{array}$$ (11)

where *y*_*t*_, *x*_*t*,*g*_, and *z*_*t*,*j*_ represent the observed values of *Y*_*t*_, *X*_*t*,*g*_, and *Z*_*t*,*g*_, respectively. Further, λ_*a*_, λ_*k*_, and λ_*q*_ are the lasso regularization parameters. The unknowns in this model, *μ*_*Y*_, *a*_*m*_, *k*_*g*_, *q*_*j*_ can be estimated from this equation by minimizing it with respect to these parameters. Once the model parameters are known, the model can be used to predict the disease case counts for the next time point.

### Kalman filtering

The Kalman filter [54] continues to be widely used for prediction and filtering problems since it is an optimal estimator in the case of linear systems which have a zero mean Gaussian measurement noise. In our context, given the measurement vector of a VAR process with added measurement noise, Kalman filter estimates the original state vector. Consider the following system of equations

$$\begin{array}{c} {\tilde{X}}_{t+1}=\tilde{A}{\tilde{X}}_{t}+W_{t+1} \end{array}$$ (12)

$$\begin{array}{c} {\tilde{Y}}_{t}=\tilde{C}{\tilde{X}}_{t}+V_{t} \end{array}$$ (13)

Here, ${\tilde{X}}_{t}$ is the state vector at time *t* of size *M* × 1, *W*_*t*_ is a zero mean Gaussian iid random *M* × 1 vector with covariance matrix *Q*, ${\tilde{Y}}_{t}$ is the observed noisy vector and *V*_*t*_ is the measurement noise characterized by a zero mean Gaussian iid random *M* × 1 vector with covariance matrix *R* that is uncorrelated with *W*_*t*_ and ${\tilde{X}}_{t}$. In our case, *Y*_*t*_ is the observed (noisy) disease case count at time *t* and *X*_*t*_ is the corresponding disease case count with the measurement noise eliminated. Further, $\tilde{A}$ and $\tilde{C}$ are matrices of sizes *M* × *M* and *M* × *m* respectively where $\tilde{C}$ has a standard known form for AR processes [50]. In our case, *A* is obtained by first fitting an AR(p) model to dengue or ILI incidence data and then converting the AR(p) model to a state-space model *A* (with *M* = *p*) using standard procedure [50]. Kalman filter can then be applied to predict ${\tilde{X}}_{t+1}$ (disease case count at time *t* + 1) given the past (noisy) disease case count ${\tilde{Y}}_{t}$.

### Ensemble methods

Ensemble methods [18] combine weighted forecasts from a set of models to come up with the final forecast. In our case, we use the additive Holt-Winters seasonal model [55] as the base model and vary different input parameters to generate multiple distinct Holt-Winters models. This is one strategy that can be used in ensemble methods. One could also use disparate models as constituent models or combine both approaches [18].

The additive Holt-Winters seasonal model [55] is given as follows:

$$\begin{array}{ccc} y_{t+1} & = & l_{t}+b_{t}+s_{t-m}\\ l_{t} & = & \alpha \left(y_{t}-s_{t-m}\right)+\left(1-\alpha \right)\left(l_{t-1}+b_{t-1}\right)\\ b_{t} & = & \beta \left(l_{t}-l_{t-1}\right)+\left(1-\beta \right)b_{t-1}\\ s_{t} & = & \gamma \left(y_{t}-l_{t}\right)+\left(1-\gamma \right)s_{t-m}. \end{array}$$ (14)

Here *y*_*t*+1_ is the forecast for time *t* + 1 and comprises three components: the level component *l*_*t*_, the trend component *b*_*t*_, and the seasonal component *s*_*t*_. Further, *m* is the length of each season (for the monthly dengue data, typically *m* = 12 and for the weekly ILI data, typically *m* = 54). The quantities *α*, *β*, and *γ* are smoothing parameters where each one ranges in value from 0 to 1. We quantify the model error using either the Root Mean Squared Error (RMSE) or Mean Absolute Relative Error (MARE) [18]. By minimizing the model error over the training data set, we can estimate the parameters *α*, *β* and *γ*.

We vary input parameters such as the season length *m*, error measure (RMSE or MARE) to be optimized for estimating the parameters, and the ending month of the training set to generate multiple (in our case, 96) distinct Holt-Winters models. Each of these models produces a monthly (weekly) forecast for the dengue (ILI) case count using data from two years preceding that month (week) as the training set. We now need to weight the forecasts of each model so that we favor the better-performing models [18]. Performance is decided as follows. Suppose we are forecasting the dengue (ILI) case count for month (week) *r* in year *v*. For each of the models, we first forecast dengue (ILI) case counts for the same month (week) *r* but in the preceding 2 years (*v* − 1 and *v* − 2). We calculate the mean forecast error *e*_*i*_ for the *i*th model using these two forecasts and the corresponding observations. The model with the largest mean forecast error e* is considered to be the worst performing model. We now compute the ratios *w*_*i*_ = e*/*e*_*i*_ for each model and this *w*_*i*_ is taken to be the weight corresponding to the *i*th model. Obviously, the best performing model (that is, the one with the smallest error) will have the highest weight. The worst performing model will have weight 1. These weights, after rounding, can be considered as votes. The *i*th method casts *round*(*w*_*i*_) votes for its forecast. We use the median value of all the votes cast as the consensus forecast of the ensemble [18]. This procedure is repeated for each monthly (weekly) forecast that is needed.

### Naive method

In this method, we use the observed value of dengue or ILI incidence at time *t* as the predicted value for dengue incidence at time *t* + 1. This sets the baseline prediction value against which other methods can be compared.

### Dengue forecast

We first apply our method to dengue case count data from five countries/states: 3 in Asia (Singapore, Taiwan, and Thailand) and 2 in South America (Brazil and Mexico). The data used is identical to the data used in [43] which has been made publicly available [56]. We consider three widely used forecast targets [57]:

- real-time dengue incidence (that is if dengue incidence data is available until time *t* − 1, we forecast dengue incidence for time *t*; this is also called nowcast),
- peak value of dengue incidence for each season (here we use the real-time dengue incidence data from above and obtain the peak value for each season within the entire time period that is forecast), and
- peak time of dengue incidence for each season (same procedure as above).

#### Setup

For our method, we choose the transformation based on an analysis of the multiplicative nature of the process as described earlier. For the other methods, *Y*_*t*_ is taken to be the log transformation of dengue case count [43] at time *t*. For all methods, we take *X*_*t*,*g*_ to be the log-transformed Google search frequency for the search term *g* at time *t*. For our method, a 4-year sliding window immediately prior to the forecast month was used for training whereas for the ARGO method a 2-year sliding window immediately prior to the forecast month was used for training, as specified in the original paper [43]. The allowed autoregressive lags used used for ARLR were months 1 to 4 and month 12.

We use a 4-year training window for our method since deseasoning is carried out by directly analyzing the time series data and a longer time window leads to a better estimate of the seasonality component that is to be removed. In ARGO and Glmnet lasso, seasonality is captured by using appropriate lags in the AR model. The choice that is made is *M* = {1, 2, …, 12, 24} months prior to estimation [43]. This is based on the hypothesis that the previous 12 months, i.e. short term influences, as well as the long term seasonal influence at 24 months which has been reported to be important for dengue prediction, are required for accurate estimation of dengue case counts. Since the lags are fixed, increasing the training window to 4 years to match with that used by ARLR method is not expected to lead to any significant changes in the results. We have verified this for the Glmnet lasso method that is a slight variant of the ARGO method. For ARGO method we have reproduced the results reported in the paper [43], which uses a 2-year training window. For Glmnet lasso, Kalman and ensemble methods, a 4-year training window was used. Since the naive method uses the observed incidence value at time *t* as the predicted incidence value at time *t*+ 1, no training period is required.

We used the dengue incidence data and Google search frequency data from [56] which is given for 5 countries/states: Singapore, Taiwan, Thailand, Brazil, and Mexico. Out-of-sample monthly estimates of dengue case counts were obtained for all methods and their performance was assessed over the following time periods [43]. Singapore: February 2008 to August 2015; Taiwan: January 2013 to March 2016; Thailand: October 2010 to August 2015; Brazil: March 2006 to December 2012; Mexico: March 2006 to August 2015.

To quantify the accuracy of the forecast, for each method we compute the following standard error measures where *e* is the forecast error vector (forecast values—actual values), *C*_*i*_ are the actual values, and *n* is the number of forecasts made:

1. RMS Error (RMSE): $\sqrt{(e\cdot e^{T})/n}$;
2. Mean Absolute Error (MAE): $1/n\sum_{i=1}^{n}\left|e_{i}\right|$;
3. Mean Absolute Percentage Error (MAPE): $1/n\sum_{i=1}^{n}\left|e_{i}\right|/C_{i}$.

From the error measures recommended in [57] (after exhaustive analysis), we have chosen the above subset that intersects with the error measures used in [43] in order facilitate direct comparison.

#### Dengue

- **Data Source**: Yang et al. [56]
- Consists of dengue case counts from five locations: Singapore, Taiwan, Thailand, Brazil and Mexico, coupled with Google trends data—top ten Google query terms in each location which are correlated with the search query ‘dengue’.
- **Error Metrics**: RMS Error, Mean Absolute Error, Mean Absolute Percentage Error
- **Forecasting Targets**: real-time dengue incidence, peak value and peak time of dengue incidence for each season

### ILI forecast

Next we apply our method to ILI case count data from the United States. The data used is identical to the data used in [44] which has been made publicly available [58]. As in the case of dengue, we consider three widely used forecast targets [57]:

- real-time ILI incidence or one-week ahead forecast (that is if ILI incidence data is available until time *t* − 1),
- peak value of ILI incidence for each season, and
- peak time of ILI incidence for each season.

In addition, we also forecast ILI incidence two, three and four weeks into the future since these forecasts are also available [44] for the ARGO method for comparison. It should be noted that these forecasts are labeled one, two and three week ahead forecasts, respectively, in ARGO since real-time ILI incidence forecast (nowcast) is labeled as zero week ahead forecast.

#### Setup

We used the ILI incidence data (CDC’s **unweighted** weekly ILI activity level), athenahealth data (weekly proportion of flu visit, ILI visit, and unspecified viral or ILI visit that are aggregated from 78,000 healthcare providers nationwide), and Google search frequency data (of 129 Google search queries most highly correlated with ILI) from [58]. Out-of-sample weekly forecasts of ILI incidence (unless otherwise specified, ILI incidence refers to unweighted ILI incidence) were obtained for the time period July 6, 2013 to February 21, 2015.

For our method and for the Kalman and ensemble methods, we took *Y*_*t*_ to be the log transformation of CDC’s unweighted ILI activity level at time *t* and *Z*_1,*t*_, *Z*_2,*t*_, and *Z*_3,*t*_ be the log transformation of weekly proportion of flu visit, ILI visit, and unspecified viral or ILI visit obtained from athenahealth data, respectively. For ARGO and Glmnet lasso methods, *Y*_*t*_ is the logit transformation of CDC’s unweighted ILI activity level at time *t* and *Z*_1,*t*_, *Z*_2,*t*_, and *Z*_3,*t*_ are the logit transformation of weekly proportion of flu visit, ILI visit, and unspecified viral or ILI visit obtained from athenahealth data. For all methods, we took the log-transformed Google Trends data. The training periods used for each method were identical to that specified above in the case of dengue. To quantify the accuracy of the forecast, for each method we compute the standard error measures described earlier.

#### ILI

- **Data Source**: Yang et al. [58]
- ILI incidence data (CDC’s unweighted weekly ILI activity level), athenahealth data (weekly proportion of flu visit, ILI visit, and unspecified viral or ILI visit 327 that are aggregated from 78,000 healthcare providers nationwide), and Google search frequency data (of 129 Google search queries most highly correlated with ILI).
- **Error Metrics**: RMS Error, Mean Absolute Error, Mean Absolute Percentage Error
- **Forecasting Targets**: real-time ILI incidence, peak value and peak time of ILI incidence for each season

## Results

### Dengue

#### Comparison of different methods

The standard forecast error measures defined above are computed for all the methods and for each country/state. The results are summarized in Tables 1–5. Rather than displaying the actual error, the ratio of the error for a given method to the error for the naive method is shown for each country. The actual error values are given in parenthesis only for the naive method. The smaller the ratio, the better the performance of corresponding method.

**Table 1:** Singapore: Realtime dengue incidence forecast error comparison.

Method	RMSE	MAE	MAPE
ARLR	0.616	0.697	0.804
ARGO	0.893	0.889	0.917
Glmnet lasso	0.734	0.765	0.826
Kalman	1.088	1.046	1.066
Ensemble	1.591	1.666	1.698
Naive	1 (340)	1 (207)	1 (0.230)

Comparison of the six methods using three different error measures. Each displayed number is the ratio of the actual error for a given method to the error for the naive method. The absolute error for the naive method is shown in parenthesis for each error measure.

**Table 2:** Taiwan: Realtime dengue incidence forecast error comparison.

Method	RMSE	MAE	MAPE
ARLR	0.398	0.445	0.401
ARGO	2.180	1.264	0.359
Glmnet lasso	1.905	1.215	0.312
Kalman	0.783	0.786	0.340
Ensemble	1.414	1.271	0.402
Naive	1 (2330)	1 (1011)	1 (1.579)

Comparison of the six methods using three different error measures. Each displayed number is the ratio of the actual error for a given method to the error for the naive method. The absolute error for the naive method is shown in parenthesis for each error measure.

**Table 3:** Thailand: Realtime dengue incidence forecast error comparison.

Method	RMSE	MAE	MAPE
ARLR	0.484	0.518	0.522
ARGO	0.715	0.715	0.706
Glmnet lasso	0.865	0.789	0.773
Kalman	0.851	0.823	0.801
Ensemble	1.464	1.457	1.927
Naive	1 (2059)	1 (1276)	1 (0.326)

Comparison of the six methods using three different error measures. Each displayed number is the ratio of the actual error for a given method to the error for the naive method. The absolute error for the naive method is shown in parenthesis for each error measure.

**Table 4:** Brazil: Realtime dengue incidence forecast error comparison.

Method	RMSE	MAE	MAPE
ARLR	0.504	0.458	0.459
ARGO	0.394	0.369	0.389
Glmnet lasso	0.784	0.596	0.511
Kalman	0.875	0.666	0.467
Ensemble	1.374	1.225	1.221
Naive	1 (30560)	1 (21678)	1 (0.546)

Comparison of the six methods using three different error measures. Each displayed number is the ratio of the actual error for a given method to the error for the naive method. The absolute error for the naive method is shown in parenthesis for each error measure.

**Table 5:** Mexico: Realtime dengue incidence forecast error comparison.

Method	RMSE	MAE	MAPE
ARLR	0.566	0.537	0.562
ARGO	0.680	0.651	0.678
Glmnet lasso	0.861	0.756	0.739
Kalman	1.035	0.899	0.809
Ensemble	1.513	1.411	1.686
Naive	1 (3570)	1 (2161)	1 (0.492)

Comparison of the six methods using three different error measures. Each displayed number is the ratio of the actual error for a given method to the error for the naive method. The absolute error for the naive method is shown in parenthesis for each error measure.

In addition to the forecast errors studied above, one could also consider errors in predicting other relevant epidemic features like peak value and peak time for each season [57]. Results for RMS errors in predicting these two quantities are shown in Tables 6 and 7. The naive method is not included since the naive method forecast is just a one time-point offset of the observational time series. Hence, for the naive method the peak value matches exactly with that of observations. Further, the peak time error for the naive method is always 1 month due to the offset. It should be noted that we do not predict the peak values and peak times at the start of the season but obtain them in a post-facto manner after we have the forecast values for the entire season. The same procedure is followed for all the methods.

**Table 6:** Dengue: Peak value forecast error comparison.

Method	Singapore	Taiwan	Thailand	Brazil	Mexico
ARLR	285	1609	1506	27781	2359
Glmnet lasso	516	5632	1714	68008	3925
Kalman	669	2164	2226	72091	7403
Ensemble	863	6323	2018	82069	10506

Comparison of the absolute RMS error for the forecast peak value of the dengue case count using four different methods for five countries. This is done post-facto as described in the text.

**Table 7:** Dengue: Peak time forecast error comparison.

Method	Singapore	Taiwan	Thailand	Brazil	Mexico
ARLR	0.707	0.577	0.000	0.000	0.471
Glmnet lasso	0.707	0.000	0.707	0.408	0.471
Kalman	4.717	1.000	1.541	4.500	1.134
Ensemble	6.325	1.915	3.381	6.764	3.780

Comparison of the absolute RMS error for the forecast peak time of the dengue case count using four different methods for five countries. This is done post-facto as described in the text.

#### Performance of ARLR method

**Fig 1:**
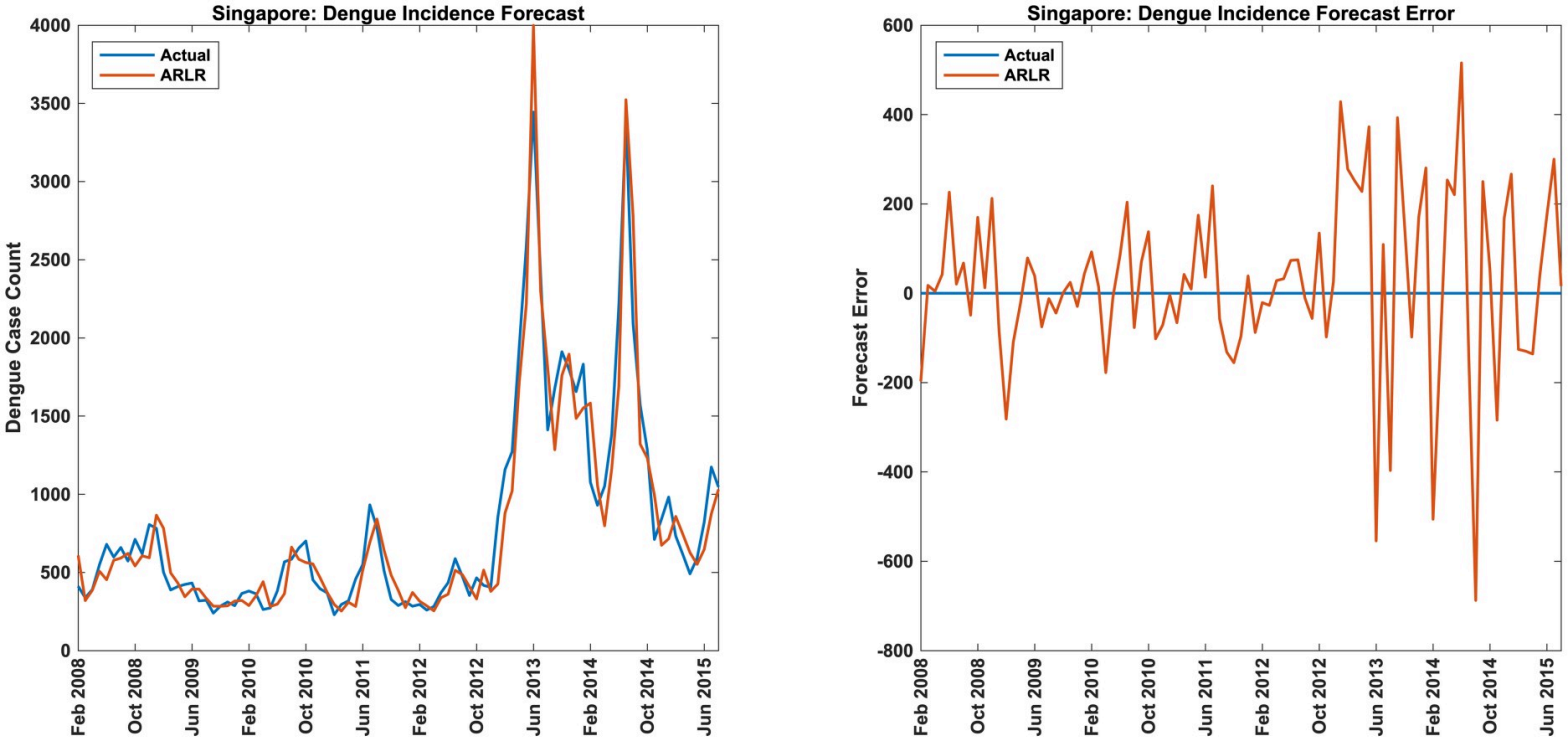
Dengue incidence forecast for Singapore. Comparison of real-time forecasts of dengue case counts with the actual values and real-time dengue forecast error (actual—predicted values) over several years for Singapore. The x-axis indicates the months starting and ending with the dates indicated.

In Fig 1 we compare the performance of real-time dengue incidence forecast using the ARLR method with the actual values for Singapore. The real-time forecast error (actual—forecast) is also plotted. Figures for the remaining countries/states can be found in the Supporting Information (S1 Fig).

### ILI

#### Comparison of different methods

The standard forecast error measures defined above are computed for all the methods. The results are summarized in Table 8. As before, rather than displaying the actual error, the ratio of the error for a given method to the error for the naive method is shown. The actual error values are given in parenthesis only for the naive method. The smaller the ratio, the better the performance of corresponding method. Results for RMS errors in predicting peak value and peak time for each season are shown in Table 9. The naive method is not included for the reasons stated earlier. As before, it should be noted that we do not predict the peak values and peak times at the start of the season but obtain them in a post-facto manner after we have the forecast values for the entire season. The same procedure is followed for all the methods. Finally the RMS errors for two-week, three-week, and four-week ahead forecasts for the top two methods (ARLR and ARGO) are compared with those for the naive method in Table 10. As usual, the ratio of the RMS error for a given method to the RMS error for the naive method is shown. The results for other error measures (MAE and MAPE) are similar.

**Table 8:** USA: Realtime ILI incidence forecast error comparison.

Method	RMSE	MAE	MAPE
ARLR	0.263	0.343	0.464
ARGO	0.315	0.403	0.481
Glmnet lasso	0.312	0.405	0.560
Kalman	1.402	1.521	1.698
Ensemble	1.380	1.241	1.165
Naive	1 (0.364)	1 (0.201)	1 (0.083)

Comparison of the six methods using three different error measures. Each displayed number is the ratio of the actual error for a given method to the error for the naive method. The absolute error for the naive method is shown in parenthesis for each error measure.

**Table 9:** ILI: Peak value and peak week forecast error comparison.

Method	Peak Value	Peak time
ARLR	0.054	0.000
Glmnet lasso	0.175	0.000
Kalman	0.557	1.144
Ensemble	0.906	1.134

Comparison of the RMS error for the forecast peak value and peak time of the ILI case count using four different methods for USA. This is done post-facto as described in the text.

**Table 10:** USA: Multi-week ahead ILI incidence forecast error comparison.

Method	2-week ahead	3-week ahead	4-week ahead
ARLR	0.285	0.386	0.453
ARGO	0.435	0.487	0.459
Naive	1 (0.607)	1 (0.759)	1 (0.873)

Comparison of the RMS error for two-week ahead, three-week ahead and four-week ahead forecasts of the ILI case count using three different methods for USA. Each displayed number is the ratio of the actual RMS error for a given method to the RMS error for the naive method. The absolute RMS error for the naive method is shown in parenthesis.

#### Performance of ARLR method

**Fig 2:**
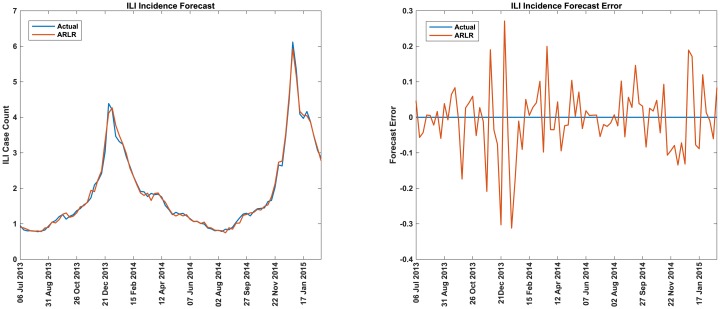
ILI incidence forecast for USA. Comparison of real-time forecasts of ILI case counts with the actual values and real-time ILI forecast error (actual—predicted values) for USA. The x-axis indicates the weeks starting and ending with the dates indicated.

**Fig 3:**
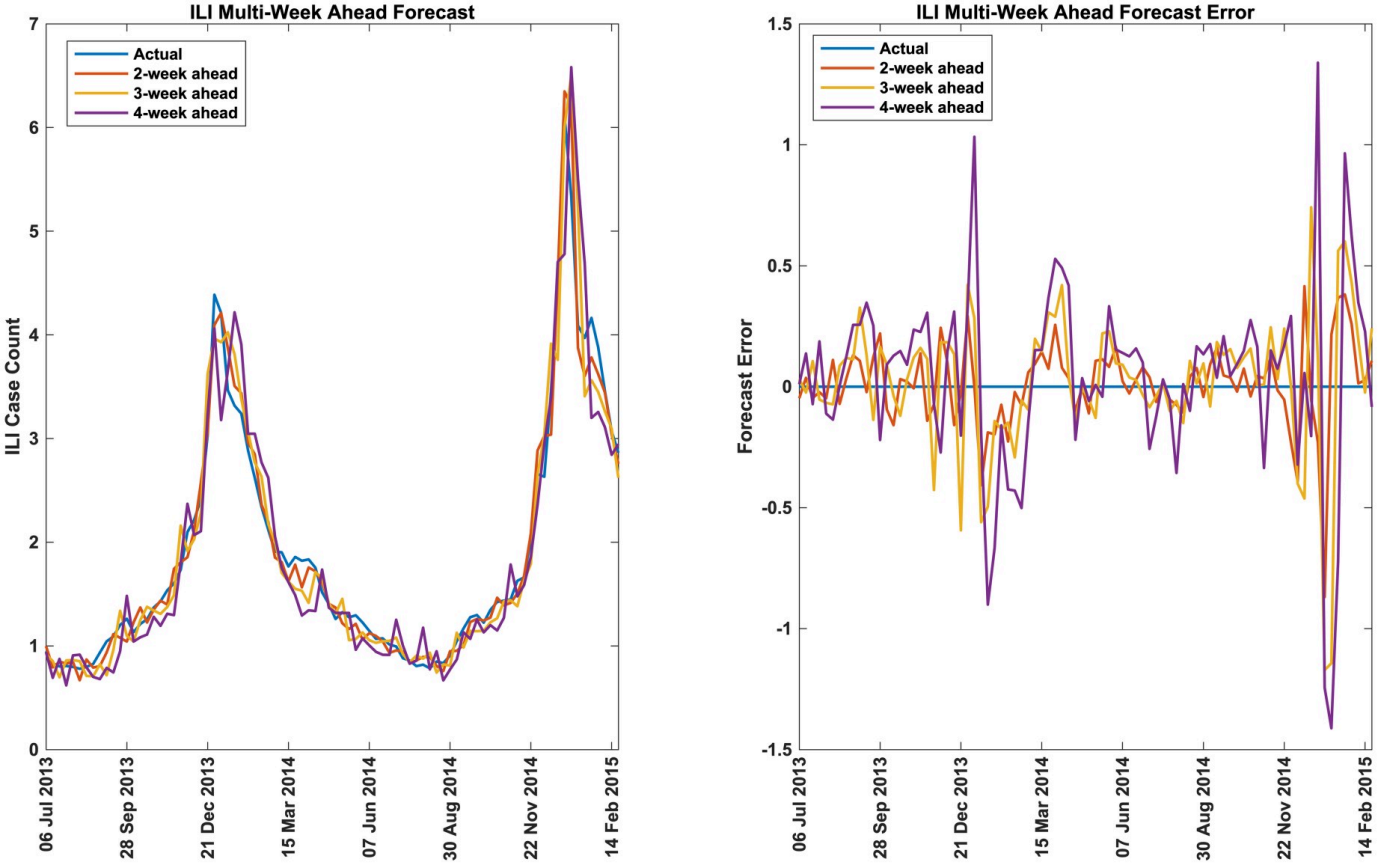
ILI incidence multi-week ahead forecast for USA. Comparison of one-week, two-week, and three-week ahead forecasts of ILI case counts with the actual values and ILI forecast error (actual—predicted values) over several years for USA. The x-axis indicates the weeks starting and ending with the dates indicated.

In Fig 2 we compare the performance of the real-time ILI incidence forecast using the ARLR method with the actual values for USA. The real-time forecast error (actual—forecast) is also plotted. In Fig 3 we compare the performance of two-week, three-week, and four-week ahead forecasts of ILI incidence using the ARLR method with the actual values for USA. The two-week, three-week, and four-week ahead forecast errors (actual—forecast) are also plotted.

### Uncertainty quantification and the effect of backfill

We quantify the uncertainties in our nowcast estimates of ILI incidence. In particular, we investigate the effect of “backfill” [47] on the forecasting accuracy. ILI incidence values are subject to backfill [47] which corresponds to a retroactive revision of ILI data as better and additional data becomes available later. In particular, the initial ILI estimates can be revised by up to ±25%. These revisions can continue for as long as 40 weeks after the publication of the initial estimates before they stabilize [47].

In order to investigate the effect of this phenomenon on our forecast accuracy, we compare the accuracies using both the revised ILI incidence data (which incorporates all the retroactive revisions that were carried out subsequent to the first reporting of the data) and the historical real-time ILI incidence data that was available [59] for each submission week of the forecasting challenge. The latter data enables us to better mimic the actual forecasting conditions [46].

In Table 11, using the standard format specified by CDC’s flu prediction challenge [60, 61], we provide the probability that the ILI incidence nowcast by ARLR method lies in the same bin (usually of width.1) as the actual value (in the Table, this row is labeled by 0). Calling this bin as the central bin, we also list the probabilities that the ILI incidence nowcast by ARLR method lies in 5 bins before the central bin (rows labeled −5 to −1) and the probabilities that the forecast lies in 5 bins after central bin (rows labeled 1 to 5). We also report the log score as defined by the CDC’s flu prediction challenge [60, 61]:

$$\begin{array}{c} \text{log}\;\text{score}=\text{log}\sum_{i=-5}^{5}p_{i}, \end{array}$$ (15)

where *p*_*i*_ is the probability that the ILI incidence nowcast by ARLR method lies in the *i*th bin as defined above and log refers to the natural logarithm. All these probabilities and log scores are listed for three different forecast weeks exhibiting a range of log scores. In order to demonstrate the effect of backfill, we replace the ILI incidences with the historical real-time ILI incidences. The probabilities and log scores for the same three forecasting weeks obtained using this historical data is shown in Table 12.

**Table 11:** ILI: Uncertainty quantification of ARLR method’s nowcast (one-week ahead forecast) using revised (backfilled) ILI data for 3 different forecast weeks.

Bin	December 28, 2013	March 14, 2014	December 20, 2014
-5	0.049	0.000	0.013
-4	0.147	0.000	0.012
-3	0.244	0.000	0.029
-2	0.142	0.000	0.062
-1	0.160	0.046	0.172
0	0.088	0.175	0.201
1	0.065	0.454	0.175
2	0.040	0.212	0.110
3	0.003	0.095	0.089
4	0.008	0.007	0.072
5	0.000	0.004	0.038
Log Score	-0.056	-0.007	-0.027

Probabilities of the ILI incidence nowcast using ARLR method lying the in the various bins defined by the CDC’s flu prediction challenge [60, 61]. The probabilities are listed for the central bin (row labeled 0) and 5 bins before and 5 bins after this central bin (rows labeled from −5 to −1 and from 1 to 5, respectively). Probabilities for three different forecast weeks are considered. The last row displays the log score as defined by the CDC’s flu prediction challenge [60, 61].

**Table 12:** Historical ILI: Uncertainty quantification of ARLR method’s nowcast (one-week ahead forecast) using historical (without backfill) ILI data for 3 different forecast weeks.

Bin	December 28, 2013	March 14, 2014	December 20, 2014
-5	0.178	0.000	0.067
-4	0.148	0.000	0.091
-3	0.126	0.000	0.130
-2	0.095	0.009	0.150
-1	0.092	0.031	0.128
0	0.029	0.157	0.099
1	0.012	0.291	0.084
2	0.000	0.288	0.036
3	0.005	0.166	0.039
4	0.002	0.048	0.022
5	0.000	0.003	0.019
Log Score	-0.3754	-0.007	-0.145

Probabilities of the ILI incidence nowcast using ARLR method lying the in the various bins defined by the CDC’s flu prediction challenge [60, 61]. The probabilities are listed for the central bin (row labeled 0) and 5 bins before and 5 bins after this central bin (rows labeled from −5 to −1 and from 1 to 5, respectively). Probabilities for three different forecast weeks are considered. The last row displays the log score as defined by the CDC’s flu prediction challenge [60, 61].

**Fig 4:**
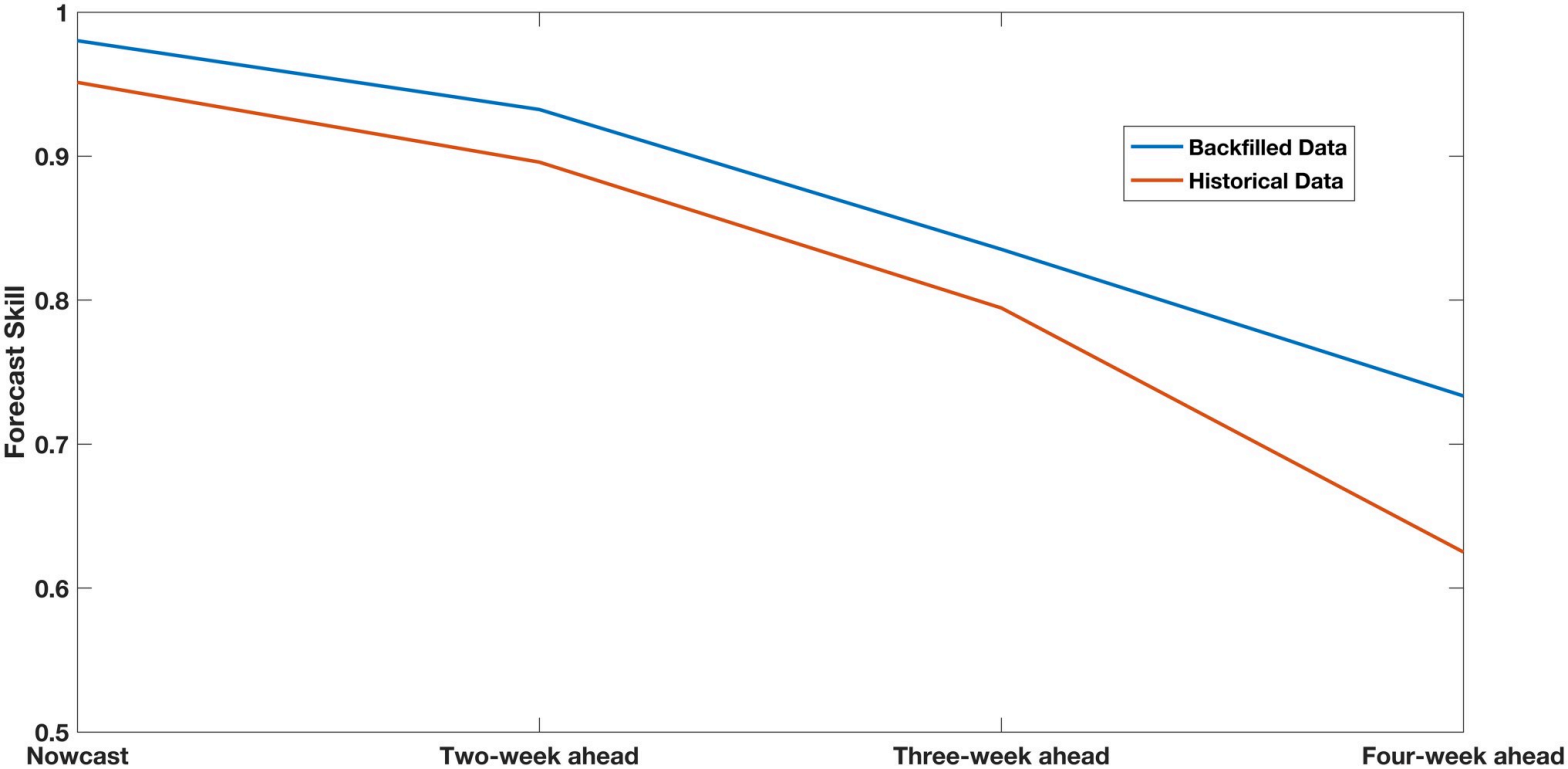
ILI: Comparison of forecast skills for forecasts using backfilled and real-time data. Comparison of forecast skills using backfilled and real-time data for nowcast, two-week, three-week, and four-week ahead forecasts.

The mean log score obtained by averaging across all forecasting windows is another quantity of interest. However, this is often reported in terms of forecast skill (or score) which is defined to be the exponential of the mean log score [42]. Forecast skills for both the backfilled and historical ILI incidence data are shown in Fig 4.

## Discussion

Forecasting disease dynamics are useful in a number of settings. This includes: planning to handle surge in hospital admissions (health care workers, protective equipment, ventilators, etc.), planning and producing pharmaceuticals, including vaccines and antivirals, taking precautionary measures such closing schools and community activities. For instance, during the 2009 H1N1 pandemic, forecasting was used for taking certain actions that included school closures in NYC. During the 2014 Ebola outbreak in West Africa, forecasts played an important role in galvanizing an international response to the outbreak. See [62, 63] for more information and discussion on this topic.

### Dengue forecasts

From the figures, it is clear that ARLR method does a good job of forecasting the dengue and ILI incidences. From Fig 3 we observe, as expected, that the forecast error increases as the forecast horizon increases. A quantitative measure of its performance is seen from the Tables. It is clearly seen that ARLR outperforms the other methods for both dengue and ILI and for all countries/states except for one (realtime dengue incidence forecast errors for Brazil). In this context it should be pointed that ARGO method (which performs better than ours in the case of Brazil) tunes the structure of the regularization parameters to optimize the results for each country/state (see the supplementary information in [43]). Our method does not require such tuning. The Glmnet lasso method is essentially identical to ARGO except that no such tuning is done. Our method does perform better than Glmnet lasso even for Brazil. Further, in all cases, ARLR forecast error is always better than the forecast error for the baseline naive method whereas this is not true for the other methods. Compared to ARGO (the next best performing method), our method (ARLR) achieves a 26% average reduction in RMSE for realtime forecast when averaged across all countries/states; a 21% reduction in MAE; and a 6% reduction in MAPE. The overall average reduction across all error measures for realtime forecast is 18% compared to the ARGO method. It should be noted that even if we use a log transformation of dengue case counts for Taiwan (as for the other countries), our method still outperforms ARGO method, but to a lesser extent. Similarly, for predicting peak value, our method achieves a 43% reduction in errors over the Glmnet lasso method when averaged across all countries/states. Predictions of peak times are also a bit better.

### ILI forecasts

For realtime (one-week ahead) ILI forecasts, ARLR method achieves a 17% reduction in RMSE, a 15% reduction in MAE, and a 3.5% reduction in MAPE as compared to ARGO. For two-week, three-week and four-week ahead ILI incidence forecasts, our method (ARLR) achieves a 19% average reduction in RMSE compared to ARGO. The substantial improvements seen using our method result from an efficient sparse representation of the time series using the Autoregressive Likelihood Ratio method and proper deseasoning of the raw time series.

RMSE of the real-time forecast for several countries for shorter window lengths of 2 and 3 years were compared with RMSE of the forecast for a window length of 4 years (that has been used in this paper). For shorter windows, the RMSE can be up to 25% higher than the RMSE for a 4-year window.

### Effect of backfill on forecast skill

Using ILI incidence data, the forecast skill for the nowcast averaged across all forecast windows is found to be 0.98 (see Fig 4). The best possible forecast skill is 1. If we use the historical ILI incidence data without backfill, we get a forecast skill of 0.95. We see that forecast skill when using backfill data can be substantially better than forecast skill obtained using realtime data (without correcting for backfill). Similar improvements are also shown in Fig 4 for two-week, three-week, and four-week ahead forecasts. Such improvements are to be expected [46, 47].

We emphasize that, even after accounting for backfill, our forecast skill values cannot be directly compared with the values obtained in the realtime CDC flu prediction challenge [60, 61] for the following reasons:

- We use unweighted ILI incidence in order to facilitate comparison with ARGO results. On the other hand, CDC’s flu prediction challenge [60, 61] uses weighted ILI incidence (where the ILI incidences are weighted with the region’s population) as the forecast target. Note that weighted ILI incidences are not simple scaled versions of the unweighted ILI incidences. In fact, weighted ILI incidence and unweighted ILI incidence can often exhibit different trends. Therefore, weighted and unweighted ILI incidences are two different forecasting targets and the corresponding forecasting errors can also be different. If we use the historical weighted ILI incidence data, we get a forecast skill of 0.90 for nowcast. This value is similar to the value reported in [45] using Dynamic Bayesian forecasting method on national data.
- We predict the ILI incidence at a national scale whereas the CDC flu prediction challenge also involves prediction at a regional scale. National scale predictions are typically better than regional scale predictions.

### Comparison across diseases and geographies

It is observed that the real-time forecast errors for ILI are smaller than those for dengue. One reason for this is the additional data source (electronic health records) that was available for forecasting ILI incidence. Across geographies, there is again variation in the errors in forecasting dengue. There could be several reasons for this such as poorer quality of the dengue incidence data collection and less widespread usage of internet.

### Comparison of different modeling approaches

Our model differs from the ARGO model in two significant ways. The ARLR method estimates the parameters using the Autoregressive Likelihood Ratio algorithm. In ARGO, the lasso method is used to estimate the parameters. The improved performance of ARLR method in forecasting dengue and ILI incidences can therefore be explained based on the comparison in the Supporting Information (S1 Text) between the Autoregressive Likelihood Ratio and lasso algorithms. In our model, the removal of the seasonal effect is carried out through a systematic process. In ARGO, this is achieved in an ad-hoc manner by incorporating lags at months 12 and 24 in the autoregressive model. Further, in ARGO, the regularization parameter is estimated using a cross-validation process. Since this is a random process, the values of the regularization parameter obtained each time are different. Hence the forecast values and the forecast error measures can differ from run to run. This drawback is absent in our method.

### Limitations

There are limitations to our method as listed below.

- Google Trend queries would be related to disease incidence in countries where a substantial proportion of the population uses internet. In developing countries with poor internet usage, such methods might not perform as well.
- Our analysis was at a national level. The data at regional scale might be statistically inferior leading to lower forecast skill.
- Our forecast skill values for ILI forecasts cannot be compared with the corresponding values obtained in the realtime CDC flu prediction challenge [60, 61] since we use unweighted ILI incidence data whereas CDC challenge uses weighted ILI incidence data.
- A recent paper [64] describes other considerations related to data preprocessing and modeling that one should be aware of while forecasting epidemics such as ILI.
- We have not used external factors such as urbanization and environmental factors such as humidity [65] and ambient temperature [66] that could play an important role.

### Future work

In our method, we have used online data such as Google Trends data and electronic health records data to facilitate comparison with the ARGO method. As directions for future work, one could also use additional sources of data such as Twitter posts, Wikipedia access logs, and crowd-sourced reporting systems [67–70]. An online forecasting system could also be implemented using our method thus enabling participation in challenges such as CDC’s flu prediction challenge [60, 61].

### Conclusions

In this paper, we have presented ARLR method for estimating a sparse autoregressive model from observations using a likelihood ratio approach. Using monthly dengue case counts and Google search term frequency data from 5 countries/states, and weekly ILI case counts, Google search term frequency data and electronic health records from USA we fitted a sparse autoregressive model (with exogenous terms) to these data using the ARLR method and obtained forecasts for dengue and ILI case counts. It was shown that the forecast error measures are, on the average, substantially lower for our method when compared to existing methods like ARGO, Glmnet lasso, Kalman filter, and ensemble method.

Our method would be most useful in cases where we need to forecast using data from multiple sources, the effect of each of which is modelled using one or more unknown parameters. In such cases, the training window immediately preceding the forecast time point has to be necessarily short since data from beyond a certain short time in the past does not contribute to the predictive ability. In summary, a large number of parameters need to be estimated using a short training window with a limited number of data points. In such cases, sparse models like ARLR become essential to obtain robust parameter estimates which then lead to more accurate forecasts. Our method could also be used to forecast incidence of other diseases.

## Supporting information

S1 Text Autoregressive Likelihood Ratio algorithm. (PDF)

S1 Fig Additional figures. Dengue incidence forecast for four countries/states. Comparison of real-time forecasts of dengue case counts and real-time dengue forecast error (actual—predicted values) over several years. The x-axis indicates the dates for each country/state.
(TIF)
