# Supplementary material for: Forecasting dengue and influenza incidences using a sparse representation of Google trends, electronic health records, and time series data
Source: PLoS Comput Biol. 2019 Nov 21;15(11):e1007518. doi: 10.1371/journal.pcbi.1007518 (PMC6894887; doi:10.1371/journal.pcbi.1007518)
Supplement: S1 Text — (PDF) [file pcbi.1007518.s001.pdf]

Supporting Information for: Forecasting dengue and influenza incidences using a sparse representation of Google trends, electronic health records, and time series data

The Autoregressive Likelihood Ratio algorithm is an example of a stepwise regression method where we start with a trivial autoregressive model and then build up a sparse model by adding only the most statistically significant variables and removing the least significant variables using a statistically principled approach. The algorithm is described below. In the subsequent section, a novel technique that enables a computationally efficient implementation of the algorithm is described. Next, Autoregressive Likelihood Ratio method is compared with the lasso method. The time complexity of the two methods are compared in the final section.

Autoregressive Likelihood Ratio Algorithm

To start, set the current VAR model, $\mathcal{M}$, to be the trivial model ($Y_t = \epsilon_t$) and set $n = 1$. Choose the set of time lags, Q, to be tested (typically Q is a finite set of consecutive integers starting from 1). Let $\mathcal{M}^{(n)}$ denote the AR equation for the nth variable $Y_t^{(n)}$ in $\mathcal{M}$. Let $S = \{Y_{t-i}^{(j)}, \quad j = 1, 2 \ldots, k; \quad i \in Q\}$ be the set of all predictor variables that are used to predict $Y_t^{(n)}$. Initialize the value p_{entry} to be equal to a small pre-chosen value p_{min}. Then for each equation $\mathcal{M}^{(n)}$, $n = 1, 2, \ldots, k$, perform the following steps:

1 For each predictor variable $x \in S$ that is not already in $\mathcal{M}^{(n)}$:

   (a) Let $\mathcal{M}_+^{(n)}$ denote the augmented equation obtained by including x in $\mathcal{M}^{(n)}$. Fit the augmented equation, $\mathcal{M}_+^{(n)}$, to the data using QR factorization.

   (b) Compute and record the log-likelihood ratio (the log-ratio of the variances of the error term of $\mathcal{M}^{(n)}$ to the error term of $\mathcal{M}_+^{(n)}$).

2 Find the largest likelihood ratio from all the ratios computed above.

3 Test if this largest ratio is greater than zero (under the null hypothesis that the ratio is zero).

4 If the p-value from the above test is less or equal to p_{entry}, change the equation $\mathcal{M}^{(n)}$ by adding to it, the corresponding variable. We now have a new current model.

5 Next, for each predictor variable that is present in the current model $\mathcal{M}^{(n)}$:

   (a) let $\mathcal{M}_-^{(n)}$ denote the contracted equation obtained by removing that predictor variable from $\mathcal{M}^{(n)}$. Fit the contracted equation, $\mathcal{M}_-^{(n)}$, to the data.

   (b) Compute and record the likelihood ratio given by the ratio of the variances of the error term of $\mathcal{M}_-^{(n)}$ to that of $\mathcal{M}^{(n)}$.

6 Find the smallest log-likelihood ratio from all the ratios computed in (v).

7 Test if this smallest ratio is greater than zero (under the null hypothesis that the ratio is zero).

8 If the p-value from the above test is greater than p_{exit}, change $\mathcal{M}^{(n)}$ by *removing* the corresponding predictor variable from $\mathcal{M}^{(n)}$. This is again the new current model.

9 Record the vector of predictor variables along with the corresponding coefficients multiplying them. Also record the fit quality for equation n using the $AICC$ (corrected AIC) criterion [52].

10 If a predictor variable has been added/removed, return to step (1).

11 If $p_{entry} < p_{max}$, increment p_{entry} by a small amount and return to step (1).

12 Use the recorded fit qualities for equation $\mathcal{M}^{(n)}$ to determine the best set of predictor variables.

The initial value, p_{min}, for the entry threshold significance level p_{entry} is chosen beforehand. p_{entry} is gradually increased (in the manner described in the algorithm) as the fit progresses until it reaches a pre-specified maximum value p_{max}. The exit threshold significance p_{exit} must satisfy the condition that $p_{exit} > p_{entry}$. We choose $p_{exit} = 2p_{entry}$. It turns out that the p_{entry} and p_{exit} values can be related to the False Discovery Rate. This provides us with a rationale to choose p_{min} and p_{max}. The value of p_{min} can be set to a "sufficiently small value"'. In theory it could be set to the smallest available double precision value. In practice, we choose the value p_{min} to be some value less than $1/(k^2q)$, where k is the number of variables in the model and q is the number of lags in the model, but choosing a smaller value has no effect other than increasing the time required for the fit.

We initially set p_{entry} to p_{min} and then gradually increase the value of p_{entry} by some multiplicative factor (say $1 + f$). The parameter p_{max} is merely some convenient upper bound on the value of p_{entry}. In case the algorithm does not terminate before p_{max} is reached, we consider the algorithm to be inappropriate for the given dataset in the sense that there is no sparse VAR representation for the dataset. For all the datasets to which we have applied this algorithm, the algorithm terminates well before p_{max} is reached.

The third parameter is the multiplicative factor $(1 + f)$ which provides the factor for increasing p_{entry}. Within a broad range, the effect of this parameter on the final model is marginal. Again, in theory, f can be chosen to be small enough so that the next model has at most one more coefficient than the previous model. In practice, we choose a value that is computationally efficient: $1 + f = \sqrt{2}$.

Efficient Implementation of the Algorithm

A naive implementation of the Autoregressive Likelihood Ratio algorithm would require solving an Ordinary Least Squares (OLS) problem for every coefficient tested at every entry and removal step. This would be prohibitively expensive. So here we describe a novel method that simultaneously tests all possible coefficients for entry into the model at every entry step. Coefficient removal, on the other hand must still be tested individually for each possible removal, but this is not so much an issue since the sparsity of the model ensures that the number of removals to be tested is comparatively small. To start with, suppose that X_0 is a matrix whose columns are predictor variables for the single vector response variable $\bar{y}$. Then the coefficient multipliers $\bar{c}_0$ for the predictors X_0 are given by the solution to:

$$X_0^T X_0 \bar{c}_0 \; = \; X_0^T \bar{y} \qquad (1)$$

Now suppose we add to the set X_0, another predictor column $\bar{x}$. Denote the extended set of predictors by

$$X = [X_0, \bar{x}]$$

The new coefficients are then given by the solution to:

$$\begin{bmatrix} X_0^T X_0 & X_0^T \bar{x} \\ \bar{x}^T X_0 & \bar{x}^T \bar{x} \end{bmatrix} \begin{bmatrix} \tilde{\bar{c}}_0 \\ c \end{bmatrix} = \begin{bmatrix} X_0^T \\ \bar{x}^T \end{bmatrix} \bar{y} \tag{2}$$

where $\tilde{\bar{c}}_0$ is the vector of modified coefficients of the predictors in X_0 and c is the coefficient of the new predictor $\bar{x}$. The vector of residuals when y is fitted to the original set of regressors X_0 is:

$$\bar{r}_0 = y - X_0 \bar{c}_0 \tag{3}$$

Set:

$$G_0 := X_0^T X_0 \tag{4}$$

$$H_0 := X_0 G_0^{-1} X_0^T \tag{5}$$

Then solving equation (2) for c and $\tilde{\bar{c}}_0$ we get:

$$c = \frac{\bar{x}^T \bar{r}_0}{\bar{x}^T (I - H_0) \bar{x}} \tag{6}$$

$$\tilde{\bar{c}}_0 = \bar{c}_0 - c\, G_0^{-1} X_0^T \bar{x} \tag{7}$$

An expression for the noise delta, which we denote by $\Delta\sigma$, can be derived by comparing the old and new residual sum of squares. We have:

$$\Delta\sigma = (y^T - \bar{c}_0^T X_0^T)(\bar{y} - X_0 \bar{c}_0) - (y^T - \tilde{\bar{c}}_0^T X_0^T - c\bar{x}^T)(\bar{y} - X_0 \tilde{\bar{c}}_0 - c\bar{x}) \tag{8}$$

Since $\bar{y} - X_0 \bar{c}_0$ and $\bar{y} - X_0 \tilde{\bar{c}}_0 - c\bar{x}$ are orthogonal, respectively, to the span of the old and new sets of regressors, this reduces to:

$$\Delta\sigma = y^T (\bar{y} - X_0 \bar{c}_0) - y^T (\bar{y} - X_0 \tilde{\bar{c}}_0 - c\bar{x}) \tag{9}$$

$$= y^T X_0 (\tilde{\bar{c}}_0 - \bar{c}_0) + y^T c\bar{x} \tag{10}$$

Using equations (2), (6) and the definitions (4) this can be written, after some matrix manipulation, as:

$$\Delta\sigma = c^2 \bar{x}^T (I - H_0) \bar{x} \tag{11}$$

In view of the above equations, computing $\bar{r}_0$ and $\bar{x}^T (I - H_0) \bar{x}$ enables us to get both c and the noise delta, while $\tilde{\bar{c}}_0$ requires us to compute the quantity $G_0^{-1} X_0^T \bar{x}$. Clearly, each of these quantities can be computed for all possible regressor extensions $\bar{x}$, simply by forming a matrix of all regressors $[\bar{x}_1, \bar{x}_2, \ldots]$ and combining each of the sequences of computations involving the $\bar{x}_i$ into a single large matrix multiplication. From the implementation point of view, this reaps huge benefits by exploiting the BLAS level 3 optimizations. Additionally, we can further simplify these computations. First, note that H_0 satisfies the following:

$$H_0 X_0 = X_0 \tag{12}$$

$$H_0 H_0^T = H_0^T H_0 = H_0 \tag{13}$$

$$H_0 = Q_0 Q_0^T \tag{14}$$

Here Q_0 is the orthonormal matrix in the QR factorization of X_0. The first two of the above equations are of general interest, while the third is directly relevant to the computation at hand. Using it we get for the quantities of interest:

$$\bar{x}^T(I - H_0)\bar{x} = \bar{x}^T\bar{x} - \bar{x}^T Q_0(\bar{x}^T Q_0)^T \quad (15)$$

$$G_0^{-1}X_0^T = R_0^{-1}Q_0^T \quad (16)$$

Here R_0 is the square upper-triangular matrix in the QR factorization of X_0. Thus the implementation hinges on computing the following:

- $\bar{x}^T\bar{x}$ which can be computed simultaneously for all regressors once and for all, at the start of the algorithm.
- R_0 via a small QR factorization (of X_0) and then $R_0^{-1}Q_0^T$ via back-substitution, at each entry step.
- $\bar{x}^T Q_0$ which can be simultaneously computed, using a single matrix multiplication for all regressors to be tested, at each entry step.

Note that for a successive run of coefficient entries without any removals, both Q_0 and $X_{test}^T Q_0$ can be simply updated by appending a column rather than recomputed (here X_{test} is the collection of all regressors to be tested).

Performance Comparison with lasso Method

We now show that our method outperforms the widely used lasso (least absolute selection and shrinkage operator) method [48,49] for fitting sparse models. This comparison is highly relevant for this paper since one of the leading methods, ARGO [43,44], for forecasting dengue and ILI incidences uses the lasso method to sparsify its model.

Lasso is a penalized or regularized version of the ordinary least squares used to solve regression problems. Consider the one-dimensional version of VAR model for simplicity:

$$Y_t = a_1 Y_{t-1} + a_2 Y_{t-2} + \cdots + a_p Y_{t-p} + \epsilon_t \quad (17)$$

where Y_t, a_i, and ϵ_t are scalars now. The goal is to estimate the parameters a_i $(i = 1, 2, \ldots, p)$ give the observed time series data y_t $(t = 1, 2, \ldots, N)$. In ordinary least squares, the parameters are estimated by minimizing the following objective function:

$$\sum_{t=p+1}^{N} (y_t - a_1 y_{t-1} - a_2 y_{t-2} - \cdots - a_p y_{t-p})^2. \quad (18)$$

In lasso [48], one adds a penalty or regularization term to the above objective function:

$$\sum_{t=p+1}^{N} (y_t - a_1 y_{t-1} - a_2 y_{t-2} - \cdots - a_p y_{t-p})^2 + \lambda \sum_{i=1}^{p} |a_i|. \quad (19)$$

The penalty term depends on a hyperparameter λ that needs to be tuned using a cross-validation process. One crucial aspect of the lasso method is the use of the l_1 norm in the regularization term instead of the standard l_2 norm used in ridge regression. The discontinuity in the first derivative of the regularization term ensures that some of the coefficients actually become zero instead of merely shrinking in a continuous way (as in ridge regression). This leads to a certain amount of sparsity in the final set of coefficients. There exist several methods to obtain a solution to (19). In this paper, for

the purpose of comparing our results with lasso, we use the `glmnet` package [1] that uses the method of coordinate descent [2].

Our method is compared with lasso by fitting VAR models using both the methods to synthetic time series data generated from sparse models. For $k = 35$ (number of variables), 25 sparse, stable, "true" models with sparsity $\approx 3\%$, and the corresponding data were randomly generated. For $k = 100$, 10 sparse, stable "true" VAR models with sparsity $\approx 1\%$ and corresponding data were generated. For each model, $N = 150$, $N = 250$, $N = 350$ and $N = 450$ (vector) time points were generated. Both the Autoregressive Likelihood Ratio and lasso fitting methods were then applied to each of the resulting data sets. The results were averaged separately over the 35 variable data set and the 100 variable dataset. The results are shown in tables 1, 2 and 3. It is seen that the number of spurious coefficients (coefficients absent in the "true" model but present in the fitted model) is more than an order of magnitude less for our method, showing that our method is better able to capture the sparsity of the underlying "true" model. The number of unpredicted coefficients (coefficients present in the "true" model but absent in the fitted model) and the RMS error for the predicted coefficients are also less for our method as compared to lasso.

(a) $k = 35$, Average over 25 VAR models

Length	150	250	350	450
glmnet	16.60	6.28	2.75	1.50
ARLR	12.99	3.75	1.72	0.88

(b) $k = 100$, Average over 10 VAR models

Length	150	250	350	450
glmnet	18.96	7.10	3.13	1.48
ARLR	15.76	5.01	1.92	0.81

Table 1. Percentage unpredicted coefficients. Results from ARLR and lasso methods are compared for different lengths N of the time series and different number of variable k.

(a) $k = 35$, Average over 25 VAR models

Length	150	250	350	450
glmnet	438.55	437.50	426.49	423.69
ARLR	14.03	10.64	9.22	9.98

(b) $k = 100$, Average over 10 VAR models

Length	150	250	350	450
glmnet	573.93	591.44	576.50	563.23
ARLR	11.14	6.18	5.72	5.69

Table 2. Percentage spurious coefficients. Results from ARLR and lasso methods are compared for different lengths N of the time series and different number of variable k.

(a) $k = 35$, Average over 25 VAR models

Length	150	250	350	450
glmnet	0.499	0.365	0.294	0.252
ARLR	0.390	0.227	0.167	0.132

(b) $k = 100$, Average over 10 VAR models

Length	150	250	350	450
glmnet	0.529	0.387	0.308	0.264
ARLR	0.416	0.246	0.166	0.123

Table 3. RMS Error in predicted coefficients. Results from ARLR and lasso methods are compared for different lengths N of the time series and different number of variable k.

Time Complexity Comparison

The time complexity of the Autoregressive Likelihood Ratio method depends on the cost of a single QR factorization that must be performed for each coefficient inclusion, the cost of the QR deletions that must be performed for each coefficient removal and the cost of certain matrix multiplications. For including a coefficient, we require just one QR factorization of a matrix of size $N \times m_{i,s}$ where $m_{i,s}$ is the number of coefficients in the equation i at step s. Let m_i be the final number of coefficients in

equation i. If we assume m_i to be an upper bound for the $m_{i,s}$ (which is approximately true in practice), then the complexity of the Autoregressive Likelihood Ratio algorithm can be shown to be:

$$T_{ARLR} \approx N \left(\sum_{i=1}^{k} (C_1 m_i^3 + C_2 m_i^2 kq) \right) \tag{20}$$

where N is the total number of time points, k is the number of variables in the model, and q is the number of lags in the model. Here we have used the fact that the theoretical time complexity for QR factorization of an $N \times m$ matrix is Nm^2 assuming that $m \leq N$. In practice, due to computational (non-algorithmic) efficiencies, the complexity is proportional to about $m^{1.6}$ rather than m^2. The constant terms C_1 and C_2 depend on the efficiency of the search path. More precisely, they are proportional to the ratio of removals to entries (we call this the reversal ratio). In tests over a variety of real and simulated data, we find that the reversal ratio (which tends to remain more or less uniform over the course of the fit process) ranges from slightly above 0 to 15. Moreover the reversal ratio increases with the density of the model.

If we assume that m_i is approximately constant for all equations (that is, $m_i \approx m \; \forall i$), the final number of coefficients M over all equations can be approximated as km. Then we may approximate the time complexity by:

$$T_{ARLR} \approx C_1 m^3 Nk + C_2 m^2 Nk^2 \tag{21}$$

The assumption of sparsity means that the average number of coefficients per equation (m) is a constant independent of the size k of the system. In addition, the number of lags is treated as a constant. Thus we may write:

$$T_{ARLR} \approx \mathcal{O}(Nk + Nk^2). \tag{22}$$

For the lasso method, it has been shown [3] that the time complexity (for a single value of the regularization parameter) is given by:

$$T_{lasso} \approx \mathcal{O}(k^3 + Nk^2). \tag{23}$$

Hence, the time complexities of the lasso method (for a single value of its regularization parameter) and our method are comparable.

However, estimating the regularization parameter in lasso typically involves fitting the model over a range of regularization parameter values. Moreover, since the regularization algorithm is non-deterministic, the value of the parameter needs to be averaged over multiple runs in lasso. Therefore, in practice, our method has a much shorter computation time as compared to lasso.

References

1. Friedman J, Hastie T, Simon N, Tibshirani R. glmnet documentation; 2015. online.

2. Friedman J, Hastie T, Tibshirani R. Regularization paths for generalized linear models via coordinate descent. Journal of Statistical Software. 2010;33(1):1–22.

3. Efron B, Hastie T, Johnstone I, Tibshirani R. Least angle regression. Annals of Statistics. 2004;32(2):407–499.
