# Supplementary figures and images for: Forecasting dengue and influenza incidences using a sparse representation of Google trends, electronic health records, and time series data
Source: PLoS Comput Biol. 2019 Nov 21;15(11):e1007518. doi: 10.1371/journal.pcbi.1007518 (PMC6894887; doi:10.1371/journal.pcbi.1007518)

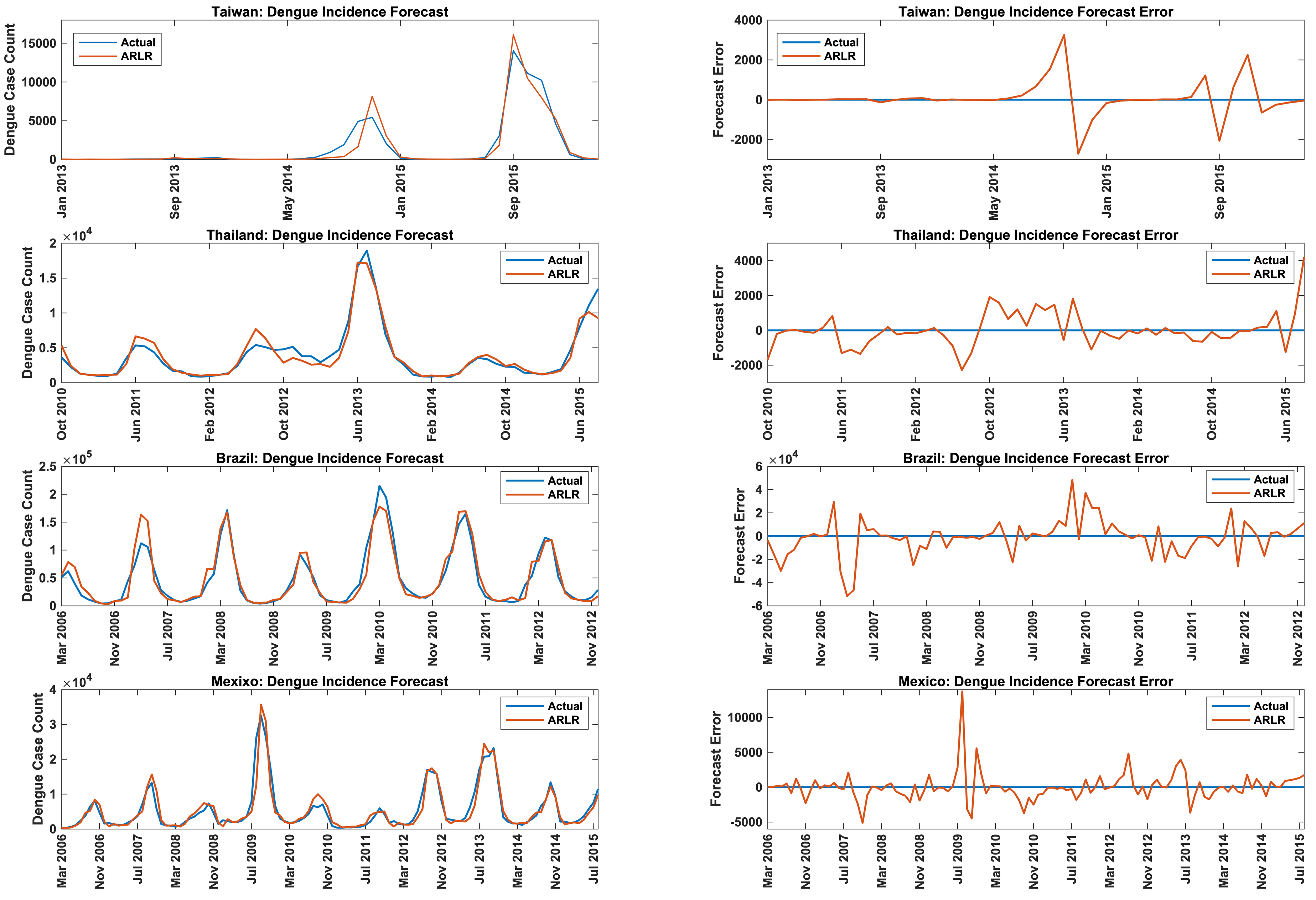

Supplement: S1 Fig — Comparison of real-time forecasts of dengue case counts and real-time dengue forecast error (actual—predicted values) over several years. The x-axis indicates the dates for each country/state. (TIF) [file pcbi.1007518.s002.tif]
